# Global Diversity and Taxonomy of *Sidera* (Hymenochaetales, Basidiomycota): Four New Species and Keys to Species of the Genus

**DOI:** 10.3390/jof7040251

**Published:** 2021-03-26

**Authors:** Zhan-Bo Liu, Meng Zhou, Yuan Yuan, Yu-Cheng Dai

**Affiliations:** Institute of Microbiology, School of Ecology and Nature Conservation, PO Box 61, Beijing Forestry University, Beijing 100083, China; zhanboliu@bjfu.edu.cn (Z.-B.L.); zhoumeng9612@bjfu.edu.cn (M.Z.)

**Keywords:** phylogenetic analysis, Rickenellaceae, wood-rotting fungi

## Abstract

The genus *Sidera* is a polypore genus with resupinate, white to cream or buff fresh basidioma, poroid or hydnoid hymenophore, a monomitic or dimitic hyphal system with generative hyphae bearing clamp connections, the presence of rosette-like crystals and allantoid to lunate basidiospores. We study the phylogeny and diversity of *Sidera* herein by using both morphological and molecular methods. Phylogenetic analyses are based on the ITS dataset, the combined 2-locus dataset (5.8S + nLSU) and 7-locus dataset (ITS + nLSU + RPB1 + RPB2 + TEF1 + mtSSU + nSSU) of 15 taxa of *Sidera* all over the world. Among them, four species are new to science and described and illustrated in this paper, viz. *S. inflata*, *S. malaysiana*, *S. punctata* and *S. roseo-bubalina.* In addition, three taxa were treated as *Sidera vulgaris* sensu lato. An identification key of the 14 accepted species of *Sidera* worldwide is provided.

## 1. Introduction

The genus *Sidera* Miettinen & K.H. Larss. (Rickenellaceae and Hymenochaetales) was established by Miettinen and Larsson [1] based on molecular and morphological analyses to accommodate *Sidera lunata* (Romell ex Bourdot & Galzin) K.H. Larss., *S. lowei* (Rajchenb.) Miettinen, *S. lenis* (P. Karst.) Miettinen and *S. vulgaris* (Fr.) Miettinen, with *S. lenis* selected as its type [1]. *Sidera* has a worldwide distribution and is characterized by white-rot, resupinate, white to cream or buff, mostly waxy fresh basidioma, poroid or hydnoid hymenophore, a monomitic or dimitic hyphal system with generative hyphae bearing clamp connections, the presence of rosette-like crystals and allantoid to lunate basidiospores [1,2].

To date, ten species are accepted in the genus, i.e., *Sidera lenis* (= *Physisporus lenis* P. Karst., Rabenhorst 1886), *S. vulgaris* (= *Polyporus vulgaris* Fr., [3]), *S. lowei* (Rajchenb.) Miettinen (= *Ceriporiopsis lowei* Rajchenb., [4]), *S. lunata* (= *Grandinia lunata* Romell ex Bourdot & Galzin, [5]), *S. minutipora* (Rodway & Cleland) Y.C. Dai et al. (= *Poria minutipora* Rodway & Cleland [6]), *S. vesiculosa* Rui Du & M. Zhou [7], *S. minutissima* Y.C. Dai et al. [2], *S. parallela* Y.C. Dai et al. [2], *S. srilankensis* Y.C. Dai et al. [2] and *S. tenuis* Y.C. Dai et al. [2].

During investigations on the diversity of polypores in tropical Asia, four resupinate polypore specimens were collected from China and Malaysia. They were characterized by a monomitic or dimitic hyphal system with generative hyphae bearing clamp connections, the presence of rosette-like crystals and allantoid to lunate basidiospores. These morphological characteristics demonstrated that these specimens may represent species of *Sidera*. To confirm their affinities, phylogenetic analyses were carried out based on the internal transcribed spacer (ITS) regions, the large subunit nuclear ribosomal RNA gene (nLSU), the largest subunit of RNA polymerase II (RPB1), the second largest subunit of RNA polymerase II (RPB2), the translation elongation factor 1-α gene (TEF1), the small subunit mitochondrial rRNA gene sequences (mtSSU) and the small subunit (nSSU) of nuclear ribosomal RNA gene. As a result, these specimens were found to represent four new terminal lineages in the *Sidera* clade. In addition, the specimens or literature and sequences of all 14 currently accepted taxa of *Sidera* were studied, with their morphological characteristics summarized in Table 1. Furthermore, an identification key of accepted species is provided.

## 2. Materials and Methods

### 2.1. Morphological Studies

Morphological descriptions were based on field notes and dry herbarium specimens. Microscopic measurements and drawings were made from slide preparations of dry tissues stained with Cotton Blue and Melzer’s reagent following Dai (2010) [10]. Pores were measured by subjectively choosing the straightest line of pores possible and measuring how many fit per mm. The following abbreviations were used: KOH = 5% potassium hydroxide; CB = Cotton Blue; CB– = acyanophilous in Cotton Blue; IKI = Melzer’s reagent; IKI– = neither amyloid nor dextrinoid in Melzer’s reagent; L = mean spore length (arithmetic average of all spores); W = mean spore width (arithmetic average of all spores); Q = variation in the L/W ratios between specimens studied; *n* (a/b) = number of spores (a) measured from given number of specimens (b). In presenting spore size variation, 5% of measurements were excluded from each end of the range, and this value is given in parentheses. Special color terms follow Anonymous (1969) and Petersen (1996) [11,12]. Herbarium abbreviations follow Thiers (2018) [13]. The studied specimens were deposited at the herbarium of the Institute of Microbiology, Beijing Forestry University (BJFC).

### 2.2. DNA Extraction, PCR and Sequencing

Total genomic DNA was extracted from dried specimens by a CTAB rapid plant genome extraction kit (Aidlab Biotechnologies Company, Limited, Beijing, China) according to the manufacturer’s instructions with some modifications [14,15]. The ITS regions were amplified with primer pairs ITS5 and ITS4 [16]. The nLSU regions were amplified with primer pairs LR0R and LR7 (https://sites.duke.edu/vilgalyslab/rdna_primers_for_fungi/ (accessed on 24 March 2021)). The mtSSU regions were amplified with primer pairs MS1 and MS2 [16]. The nSSU regions were amplified with primer pairs NS1 and NS4 [16]. The TEF1 regions were amplified with primer pairs EF1-983F and EF1-1567R [17]. The RPB1 regions were amplified with primer pairs RPB1-Af and RPB1-Cf [18]. The RPB2 regions were amplified with primer pairs fRPB2-f5F and bRPB2-7.1R [19].

The PCR procedure for ITS and mtSSU was as follows: initial denaturation at 95 °C for 3 min, followed by 34 cycles at 94 °C for 40 s, 54 °C for ITS and 55 °C for mtSSU for 45 s and 72 °C for 1 min, and a final extension of 72 °C for 10 min. The PCR procedure for nLSU, nSSU and TEF1 was as follows: initial denaturation at 94 °C for 1 min, followed by 34 cycles at 94 °C for 30 s, 50 °C for nLSU and 59 °C for TEF1 for 1 min and 72 °C for 1.5 min, and a final extension of 72 °C for 10 min. The PCR procedure for RPB1 and RPB2 was as follows: initial denaturation at 94 °C for 2 min, followed by 10 cycles at 94 °C for 40 s, 60 °C for 40 s and 72 °C for 2 min, then followed by 37 cycles at 94 °C for 45 s, 55 °C for 1.5 min and 72 °C for 2 min, and a final extension of 72 °C for 10 min. The PCR products were purified and sequenced in the Beijing Genomics Institute, China, with the same primers used in the PCR reactions.

### 2.3. Phylogenetic Analyses

Three combined matrices were reconstructed for phylogenetic analyses as an ITS dataset, a two-gene dataset (5.8S + nLSU) and a 7-gene dataset (ITS + nLSU + RPB1 + RPB2 + TEF1 + mtSSU + nSSU). Phylogenetic analyses were performed with maximum likelihood (ML) and Bayesian Inference (BI) methods. Sequences generated in this study were aligned with additional sequences downloaded from GenBank (Table 2) in MAFFT 6 ([20]; http://mafft.cbrc.jp/alignment/server/ (accessed on 24 March 2021)) using the “G–INS–i” strategy and manually adjusted in BioEdit [21]. Prior to phylogenetic analysis, ambiguous sequences at the start and the end were deleted, and gaps were manually adjusted to optimize the alignment. The data matrix was edited in Mesquite v3.04 software [22]. Sequence alignment was deposited at TreeBase (submission ID 27909, 27910 and 27911). Sequences of *Exidia candia* Lloyd and *Exidiopsis calcea* (Pers.) K. Wells outside Hymenochaetales were used as the outgroup [1,12,23] in the combined 2-gene dataset (5.8S + nLSU) and 7-gene dataset (ITS + nLSU + RPB1 + RPB2 + TEF1 + mtSSU + nSSU). The sequence of *Sidera lunata* (Romell ex Bourdot & Galzin) K.H. Larss. was used as the outgroup in the ITS dataset.

Sequences were also analyzed using maximum likelihood (ML) with RAxML-HPC through the CIPRES Science Gateway ([24]; http://www.phylo.org (accessed on 24 March 2021)). Statistical support values (BS) were obtained using nonparametric bootstrapping with 1000 replicates. The optimal substitution models for the combined dataset were determined using the Akaike Information Criterion (AIC) implemented in MrModeltest 2.2 [25] after scoring 24 models of evolution by PAUP* version 4.0 beta 10 [26]. The selected model applied in the Bayesian phylogenetic inference (BI) analyses and maximum likelihood (ML) analyses was the model GTR + I + G.

The BI analysis was performed with MrBayes 3.2.5 [27]. Four Markov chains were run for two runs from random starting trees for 3 million generations (ITS, 5.8S + nLSU and ITS + nLSU + RPB1 + RPB2 + TEF1 + mtSSU + nSSU) until the split deviation frequency value reached <0.01, and trees were sampled every 1000 generation. The first 25% of the sampled trees were discarded as burn-in, and the remaining ones were used to reconstruct a majority rule consensus tree and calculate Bayesian posterior probabilities (BPP) of the clades.

Branches that received bootstrap support for maximum likelihood (BS), and Bayesian posterior probabilities (BPP) greater than 70% (BS) and 0.95 (BPP) were considered as significantly supported, respectively. FigTree v1.4.2 [28] was used to visualize the resulting tree.

## 3. Results

### 3.1. Phylogenetic Analyses

**Figure 1:**
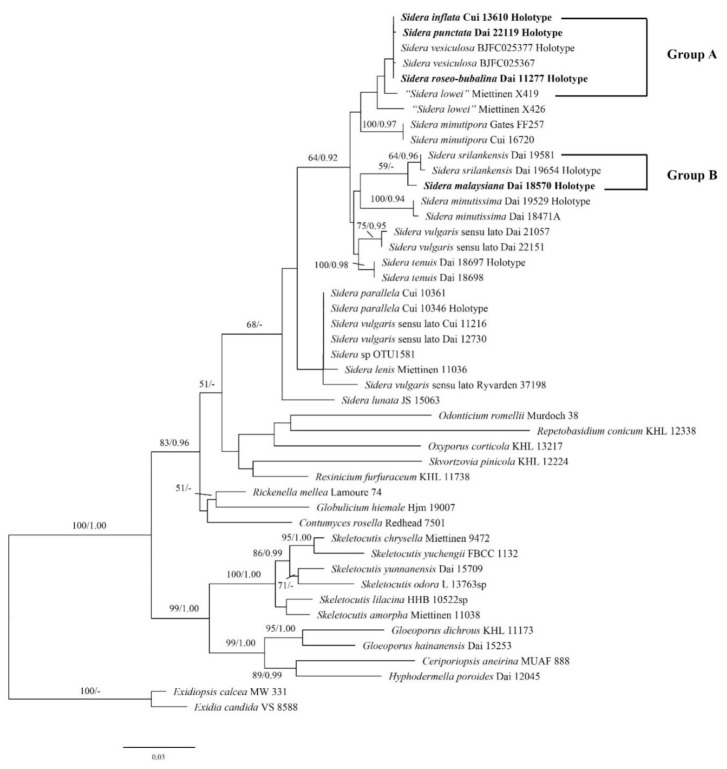
Phylogeny of *Sidera* and related species generated by ML analyses based on combined 5.8S + nLSU sequences. Branches are labeled with maximum likelihood bootstrap >50% and Bayesian posterior probabilities >0.90, respectively. New species are indicated in bold.

**Figure 2:**
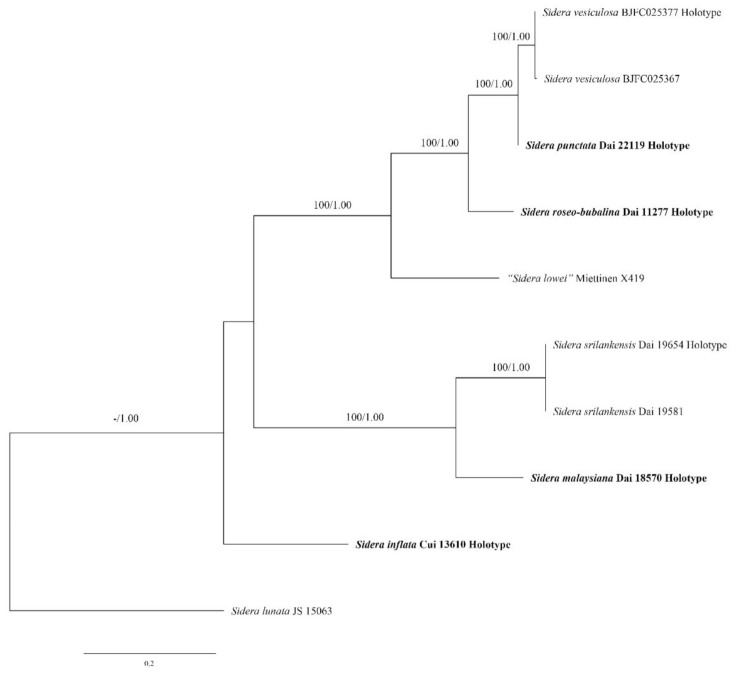
Phylogeny of *Sidera* new species and related species generated by ML analyses based on ITS sequences. Branches are labeled with maximum likelihood bootstrap >50% and Bayesian posterior probabilities >0.90, respectively. New species are indicated in bold.

ITS is the most important locus for barcoding fungi, especially at the generic and species levels. However, the genetic variation inside *Sidera* is significant and, for example, the alignment of the whole ITS region is difficult among *Sidera* polypores [1]. Therefore, we used the most stable and conservative portion of ITS (5.8S) and LSU to analyse the phylogenetic relationship of *Sidera* species (Figure 1). The concatenated 5.8S + nLSU dataset contained sequences from 46 fungal specimens representing 15 *Sidera* taxa (three are treated as *S. vulgaris* sensu lato; (Table 2). Sequences of *Exidia candia* and *Exidiopsis calcea* were used as the outgroup [1,12,23]. The best model for the 5.8S + nLSU dataset estimated and applied in the Bayesian analysis was GTR + I+G. The ML analysis resulted in the best tree (Figure 1). BI analyses resulted in almost identical tree topologies compared to the ML analysis, with an average standard deviation of split frequencies of 0.009930 (BI). Additionally, only the ML tree is presented along with the support values from the BI analyses. Our new species and their related species are nested in Group A and Group B. Thus, we used the whole ITS region to analyse the phylogenetic relationship of new *Sidera* species with their closely related species (Figure 2).

The ITS dataset contained sequences from ten fungal specimens representing eight *Sidera* taxa (four new species and another four *Sidera* taxa). The sequence of *Sidera lunata* was used as the outgroup. The best model for the ITS dataset estimated and applied in the Bayesian analysis was GTR + I + G. The ML analysis resulted in the best tree (Figure 2). BI analyses resulted in almost identical tree topologies compared to the ML analysis, with an average standard deviation of split frequencies of 0.001361 (BI). Thus, only the ML tree is presented along with the support values from the BI analyses.

**Figure 3:**
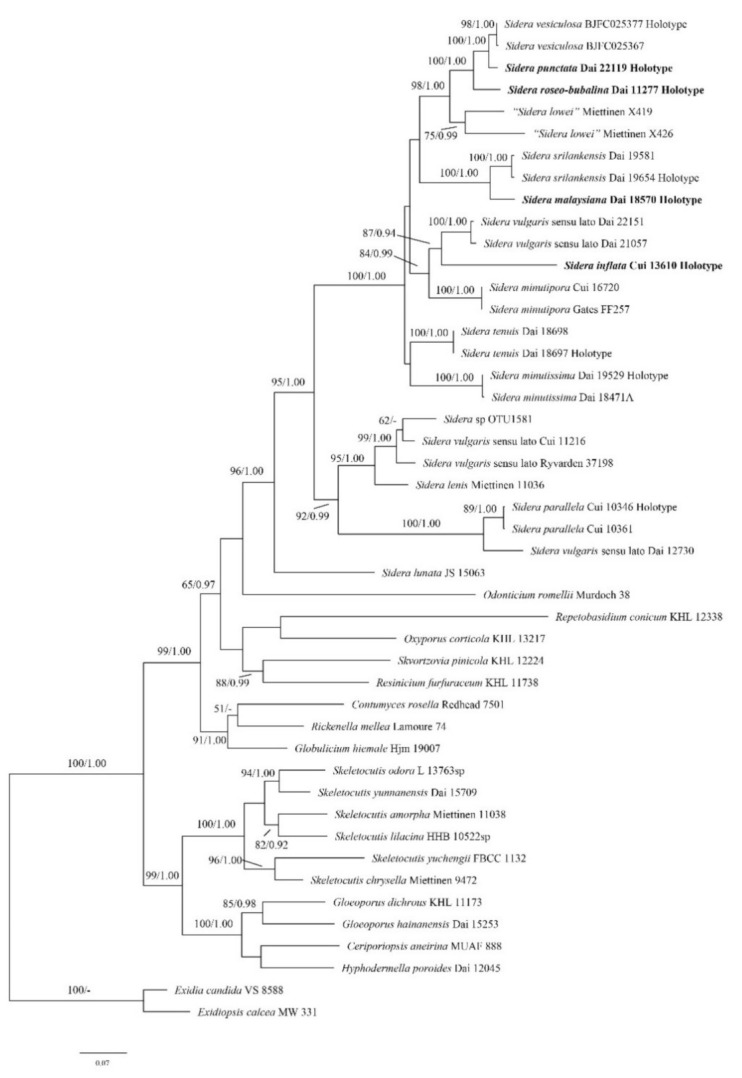
Phylogeny of *Sidera* and related species generated by ML analyses based on combined ITS + nLSU + RPB1 + RPB2 + TEF1 + mtSSU + nSSU sequences. Branches are labeled with maximum likelihood bootstrap >50% and Bayesian posterior probabilities >0.90, respectively. New species are indicated in bold.

The concatenated ITS + nLSU + RPB1 + RPB2 + TEF1 + mtSSU + nSSU dataset contained sequences from 46 fungal specimens representing 15 *Sidera* taxa (three were treated as *S. vulgaris* sensu lato; Table 2). Sequences of *Exidia candia* and *Exidiopsis calcea* were used as the outgroup [1,12,23]. The best model for the ITS + nLSU + RPB1 + RPB2 + TEF1 + mtSSU + nSSU dataset estimated and applied in the Bayesian analysis was GTR + I+G. The ML analysis resulted in the best tree (Figure 3). BI analyses resulted in almost identical tree topologies compared to the ML analysis, with an average standard deviation of split frequencies of 0.009821 (BI). Additionally, only the ML tree is presented along with the support values from the BI analyses.

The phylogenetic trees (Figure 2 and Figure 3) revealed four new and independent lineages represented by our specimens, indicating that they are phylogenetically distinct from the species currently known in the genus. In addition, another three taxa were treated as *S. vulgaris* sensu lato, although they formed four independent lineages too.

### 3.2. Taxonomy

**Figure 4:**
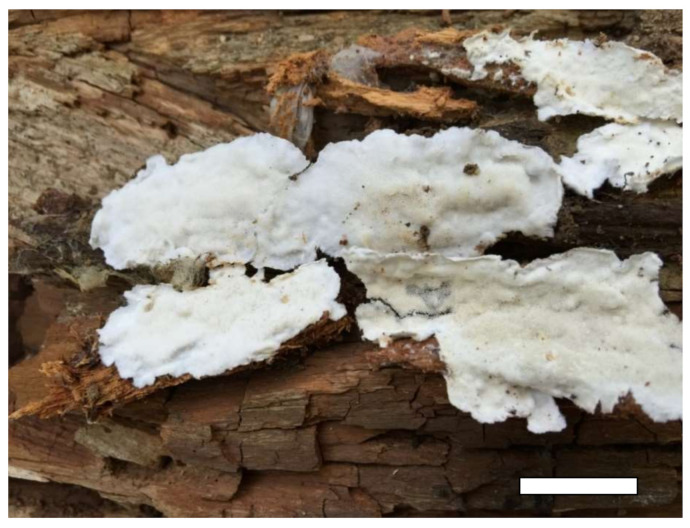
A basidioma of *Sidera inflata* (from the holotype Cui 13610). Scale bar = 10 mm.

**Figure 5:**
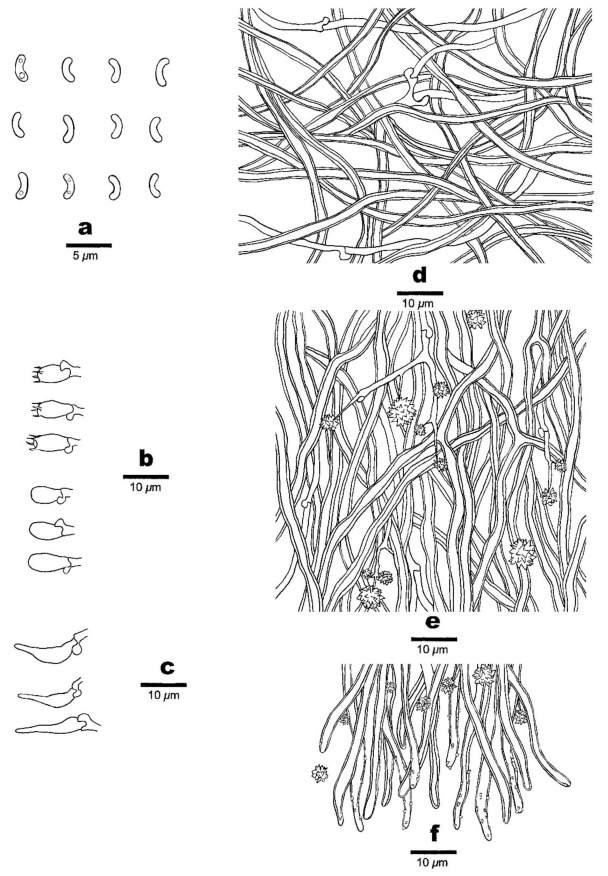
Microscopic structures of *Sidera inflata* (holotype, Cui 13610). (**a**) Basidiospores. (**b**) Basidia and basidioles. (**c**) Cystidioles. (**d**) Hyphae from subiculum. (**e**) Hyphae from trama. (**f**) Hyphae at dissepiment edge. Drawing by Meng Zhou.

1. ***Sidera inflata*** Z.B. Liu & Y.C. Dai, sp. nov. Figure 4 and Figure 5.

MycoBank number: MB 838380.

Etymology—*Inflata* (Lat.): referring to the species having inflated skeletal hyphae in KOH.

Type—China, Hainan Province, Baisha County, Yinggeling Nature Reserve, on rotten angiosperm wood, 17 November 2015, B.K. Cui 13610 (holotype BJFC 028475).

Basidioma—Annual, resupinate, soft corky when fresh and dry, up to 4 cm long, 1.5 cm wide and less than 1 mm thick at center; pore surface white to buff and shiny when fresh, becoming cream to buff yellow and shiny when dry; sterile margin distinct, white, cottony, thinning out; pores angular, 9–10 per mm; dissepiments thin, lacerate; subiculum very thin to almost absent; tubes concolorous with the poroid surface, less than 1 mm long.

Hyphal structure—Hyphal system dimitic; generative hyphae bearing clamp connections; skeletal hyphae dominant; all hyphae IKI–, CB–, skeletal hyphae obviously becoming swollen in KOH.

Subiculum—Generative hyphae hyaline, thin-walled, occasionally branched, 1–2 μm in diameter; skeletal hyphae thick-walled with a wide lumen, rarely branched, interwoven, 2–4 μm diameter; rosette-like crystals rarely present.

Tubes—Generative hyphae hyaline, thin-walled, occasionally branched, 1–2 μm in diameter; skeletal hyphae thick-walled with a wide lumen, occasionally branched, interwoven, usually covered by abundant fine thorn-like crystals at dissepiment edge, 2–4 μm diameter; rosette-like crystals abundant, 2–8 μm in diameter; cystidia absent; cystidioles present, fusoid, hyaline, thin-walled, basally swollen, with a sharp or often hyphoid neck, 13–15 × 2.5–3 μm; basidia clavate, hyaline, bearing four sterigmata and a basal clamp connection, 7–8 × 3.5–4.5 μm; basidioles similar in shape to basidia, but slightly shorter.

Basidiospores—Allantoid, hyaline, thin-walled, smooth, occasionally with one or two guttules, IKI–, CB–, (2.9–)3–3.3(–3.5) × (0.8–)0.9–1.1 μm, L = 3.03 μm, W = 1 μm, Q = 3.03 (*n* = 60/1).

Notes—*Sidera inflata* was found in China, and the species is characterized by annual, resupinate basidioma with a white to buff fresh pore surface, which becomes cream to buff-yellow upon drying; angular pores (9–10 per mm); a dimitic hyphal system; skeletal hyphae at dissepiment edge bearing abundant fine thorn-like crystals; skeletal hyphae in all structures obviously swelling in KOH; and allantoid basidiospores measuring 3–3.3 × 0.9–1.1 μm. Morphologically, *S. inflata* can be distinguished from other species in *Sidera* by its skeletal hyphae at the dissepiment edge, which are usually covered by abundant fine thorn-like crystals. Although *S. inflata* clustered together with the *S. vulgaris* sensu lato (Dai 21057 and Dai 22151) with a moderate support (87% BS, 0.94 BPP; Figure 3), the latter taxon had perennial basidioma, and its skeletal hyphae were unchanged in KOH.

**Figure 6:**
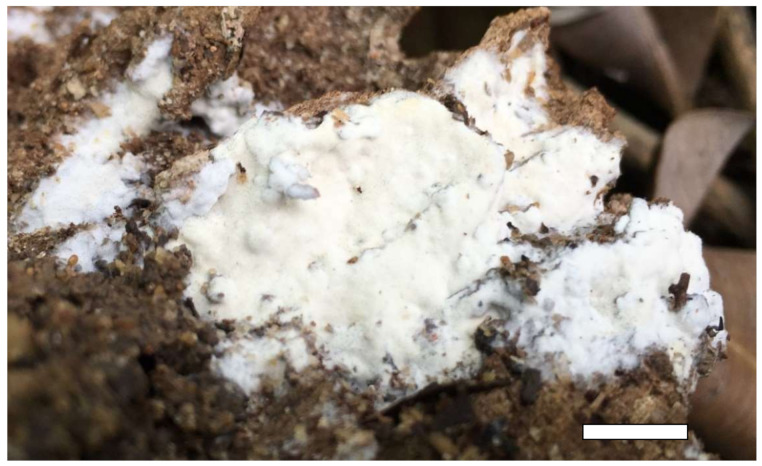
A basidioma of *Sidera malaysiana* (from the holotype Dai 18570). Scale bar = 10 mm.

**Figure 7:**
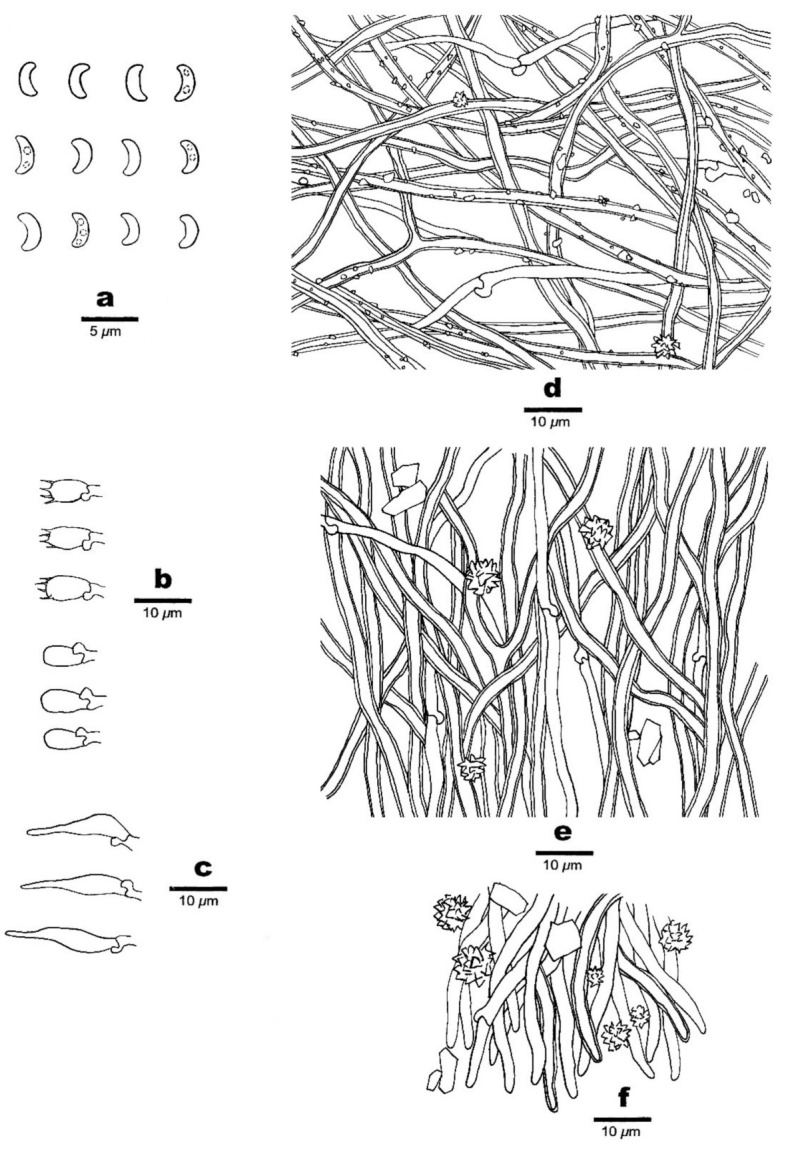
Microscopic structures of *Sidera malaysiana* (holotype, Dai 18570). (**a**) Basidiospores. (**b**) Basidia and basidioles. (**c**) Cystidioles. (**d**) Hyphae from subiculum. (**e)** Hyphae from trama. (**f**) Hyphae at dissepiment edge. Drawing by Meng Zhou.

2. ***Sidera malaysiana*** Z.B. Liu & Y.C. Dai, sp. Nov. Figure 6 and Figure 7

MycoBank number: MB 838381.

Etymology—*Malaysiana* (Lat.): referring to the species occurring in Malaysia.

Type—Malaysia, Selangor, Forest Research Institute of Malaysia, on rotten angiosperm wood, 15 April 2018, Y.C. Dai 18570 (holotype BJFC 026859).

Basidioma—Annual, resupinate, very difficult to separate from substrate, soft corky when fresh and dry, up to 4 cm long, 2 cm wide and less than 1 mm thick at center; pore surface white to cream when fresh and dry; sterile margin indistinct; pores round to angular, 9–11 per mm; dissepiments thin, entire; subiculum very thin to almost absent; tubes white, less than 1 mm long.

Hyphal structure—Hyphal system dimitic; generative hyphae bearing clamp connections; skeletal hyphae dominant; all hyphae IKI–, CB–, skeletal hyphae slightly swollen in KOH.

Subiculum—Generative hyphae hyaline, thin-walled, rarely branched, 1–2 μm in diameter; skeletal hyphae dominant, occasionally branched, interwoven, usually covered by abundant irregular crystals and fine thorn-like crystals, 1.5–3.5 μm diameter; rosette-like crystals occasionally present, 3–5 μm in diameter.

Tubes—Generative hyphae hyaline, thin-walled, rarely branched, 1–2.5 μm in diameter, dominating at dissepiment edges; skeletal hyphae thick-walled, occasionally branched, interwoven, 1.5–3.5 μm diameter; rosette-like and irregular rhomboidal crystals abundant at dissepiment edges; cystidia absent; cystidioles present, fusoid, hyaline, thin-walled, basally swollen, with a sharp or often hyphoid neck, 9–13 × 2.2–3.5 μm; basidia clavate, hyaline, with a basal clamp connection and four sterigmata, 7.8–15 × 3–4.3 μm; basidioles similar in shape to basidia, but slightly shorter.

Basidiospores—Lunate, hyaline, thin-walled, smooth, usually with two or three guttules, IKI–, CB–, (2.8–)2.9–3.2(–3.3) × 1–1.2(–1.4) μm, L = 3.16 μm, W = 1.12 μm, Q = 2.82 (*n* = 60/1).

Notes—*Sidera malaysiana* was found in Malaysia, and the species is characterized by annual, resupinate basidioma with a white to cream pore surface, round to angular pores (9–11 per mm), a dimitic hyphal system, skeletal hyphae in all structures become slightly swollen in KOH, subicular skeletal hyphae bearing rosette-like crystals, irregular crystals and fine thorn-like crystals and lunate basidiospores measuring 2.9–3.2 × 1–1.2 μm. Morphologically, *S. malaysiana* can be distinguished from other species in *Sidera* by its subicular skeletal hyphae, which are usually covered by abundant irregular crystals and fine thorn-like crystals. *S. malaysiana* is closely related to *S. srilankensis* in our phylogeny (100% BS, 1.00 BPP; Figure 2), but *S. malaysiana* is different from *S. srilankensis* due to its smaller pores (9–11 per mm vs. 6–8 per mm, [2]) and skeletal hyphae in all structures becoming slightly swollen in KOH, while skeletal hyphae are unchanged in KOH in *S. srilankensis. S. malaysiana* resembles *S. parallela* due to its white fresh pore surface. However, *S. malaysiana* differs from *S. parallela* due to its smaller pores (9–11 per mm vs. 6–8 per mm, [2]). In addition, tramal hyphae are parallel in *S. parallela*, while they are interwoven in *S. malaysiana*. Additionally, they are phylogenetically distant (Figure 2).

**Figure 8:**
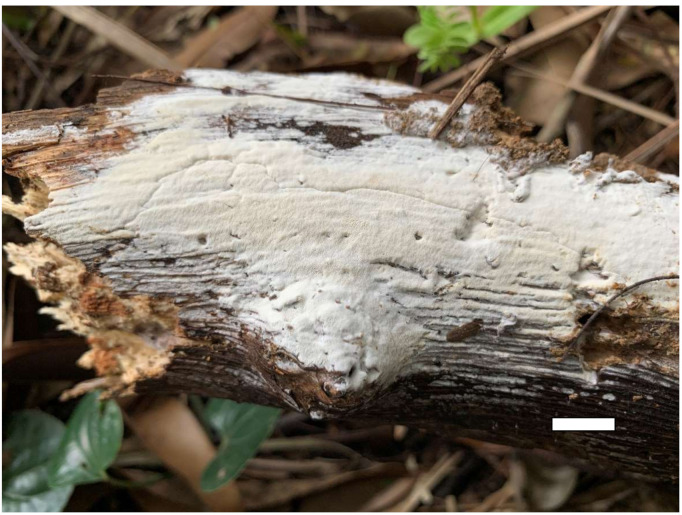
A basidioma of *Sidera punctata* (from the holotype Dai 22119). Scale bar = 10 mm.

**Figure 9:**
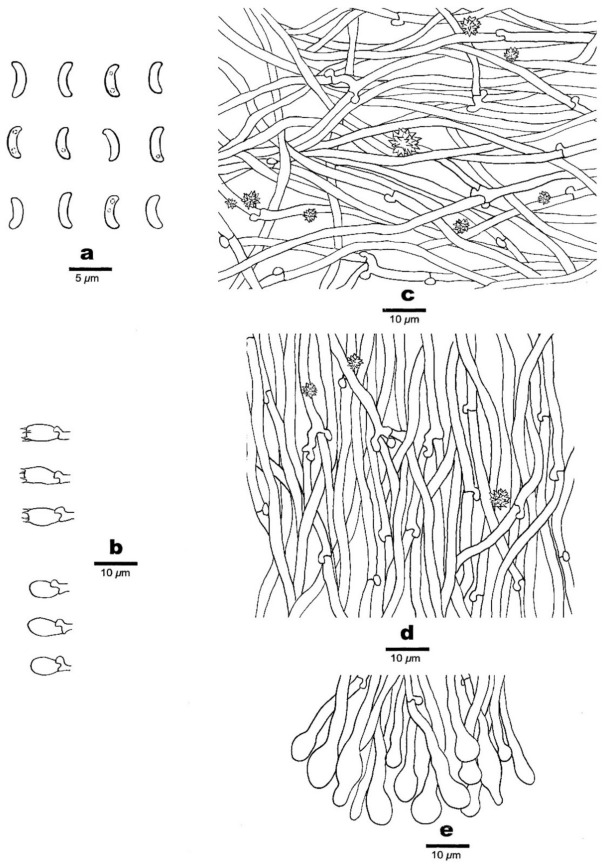
Microscopic structures of *Sidera punctata* (holotype, Dai 22119). (**a**) Basidiospores. (**b**) Basidia and basidioles. (**c**) Hyphae from subiculum. (**d**) Hyphae from trama. (**e**) Hyphae at dissepiment edge. Drawing by Meng Zhou.

3. ***Sidera punctata*** Z.B. Liu & Y.C. Dai, sp. nov. Figure 8 and Figure 9.

MycoBank number: MB 838384.

Etymology—*Punctata* (Lat.): referring to the species having cushion-shaped basidioma.

Type—China, Hainan Province, Haikou, Guanlanhu, on rotten angiosperm wood, 18 November 2020, Y.C. Dai 22119 (holotype BJFC 036011).

Basidioma—Annual, resupinate, soft corky when fresh and dry, up to 13 cm long, 4 cm wide and less than 1 mm thick at center; pore surface white to cream when fresh, becoming cinnamon buff to white when dry; sterile margin distinct, white, cottony, thinning out; pores round, 8–9 per mm; dissepiments thin, entire; subiculum very thin to almost absent; tubes darker than the poroid surface, less than 1 mm long.

Hyphal structure—Hyphal system monomitic; generative hyphae bearing clamp connections; all hyphae IKI–, CB–, unchanged in KOH.

Subiculum—Generative hyphae hyaline, thin-walled, often branched, interwoven, 1.5–2.5 μm in diameter; rosette-like crystals frequently present, 2–7 μm in diameter.

Tubes—Generative hyphae hyaline, thin-walled, sometimes branched, loosely interwoven, 1.5–3 μm in diameter, some hyphae, especially hyphae at dissepiment edge with swollen tips which are globose, bottle-shaped or irregularly elongated; rosette-like crystals occasionally present; cystidia absent; cystidioles absent; basidia clavate, hyaline, bearing four sterigmata and a basal clamp connection, 12.5–13.5 × 3.5–4.5 μm; basidioles similar in shape to basidia, but slightly shorter.

Basidiospores—Allantoid to lunate, hyaline, thin-walled, smooth, occasionally with one to three guttules, IKI–, CB–, (3.5–)3.8–4.8(–5) × 1–1.3(–1.4) μm, L = 4.27 μm, W = 1.21 μm, Q = 3.53 (*n* = 60/1).

Notes—*Sidera punctata* was discovered in China, and the species is characterized by annual, resupinate basidioma with a white to cream fresh pore surface which becomes cinnamon-buff to white upon drying, round pores (8–9 per mm), a monomitic hyphal system, and allantoid to lunate basidiospores measuring 3.8–4.8 × 1–1.3 μm. Phylogenetically, *S. punctata* is close to *S. vesiculosa* (100% BS, 1.00 BPP; Figure 2) and it is similar to *S. vesiculosa* by annual, resupinate basidioma and some generative hyphae with swollen tips, but *S. punctata* has a cinnamon-buff to white dry pore surface, while the pore surface is cream upon drying in *S. vesiculosa*. Above all, *S. punctata* can be distinguished from other species in *Sidera* by its rosette-like crystals are more abundant in subiculum than in tubes, while rosette-like crystals are more abundant in tubes than in subiculum in other members of the genus.

**Figure 10:**
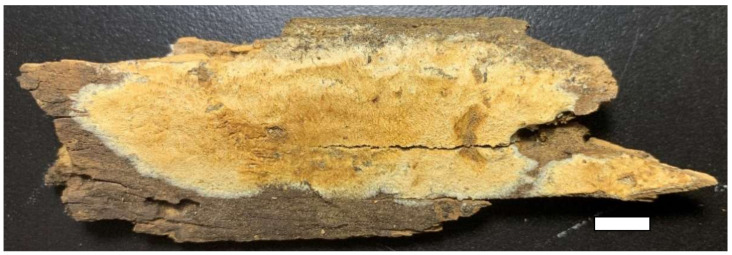
A dry basidioma of *Sidera roseo-bubalina* (from the holotype Dai 11277). Scale bar = 10 mm.

**Figure 11:**
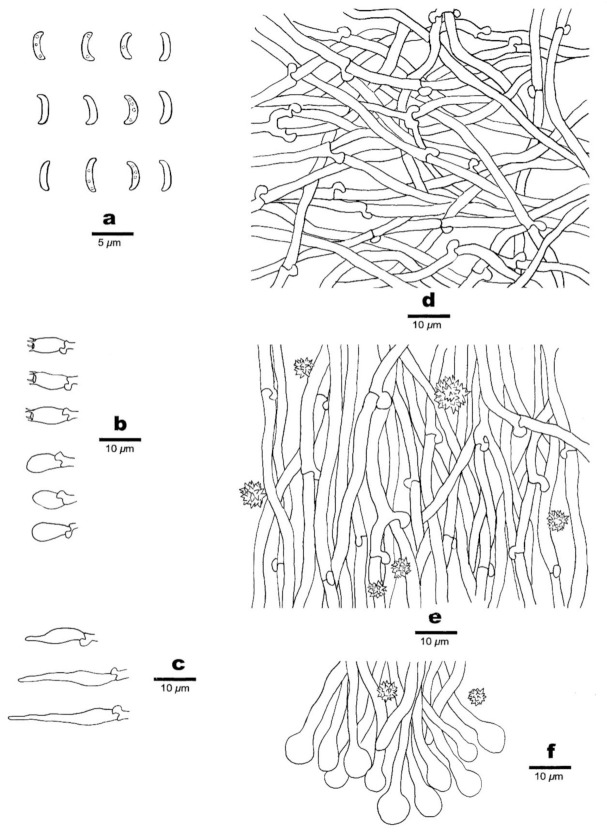
Microscopic structures of *Sidera roseo-bubalina* (holotype, Dai 11277). (**a**) Basidiospores. (**b**) Basidia and basidioles. (**c**) Cystidioles. (**d**) Hyphae from subiculum. (**e**) Hyphae from trama. (**f**) Hyphae at dissepiment edge. Drawing by Meng Zhou.

4. ***Sidera roseo-bubalina*** Z.B. Liu & Y.C. Dai, sp. nov. Figure 10 and Figure 11.

MycoBank number: MB 838382.

Etymology—*Roseo-bubalina* (Lat.): referring to the species having pinkish buff hymenophore.

Type—China, Henan Province, Neixiang County, Baotianman Nature Reserve, on rotten wood of *Quercus*, 22 September 2009, Y.C. Dai 11277 (holotype BJFC 007251).

Basidioma—Annual, resupinate, soft corky when dry, up to 7 cm long, 3 cm wide, and less than 1 mm thick at center; pore surface pinkish buff to yellowish brown when dry; sterile margin distinct, white, cottony, thinning out; pores round, 6–7 per mm; dissepiments thin, entire to lacerate; subiculum very thin to almost absent; tubes concolorous with the poroid surface, less than 1 mm long.

Hyphal structure—Hyphal system monomitic; generative hyphae bearing clamp connections; all hyphae IKI–, CB–, unchanged in KOH.

Subiculum—Generative hyphae hyaline, thin-walled, often branched, interwoven, 2–3 μm in diameter; rosette-like crystals occasionally present.

Tubes—Generative hyphae hyaline, thin-walled, often branched, interwoven, 2–3.5 μm in diameter, some hyphae, especially hyphae at dissepiment edge with swollen tips which are globose, bottle-shaped or irregularly elongated; rosette-like crystals abundant, 4–6 μm in diameter; cystidia absent; cystidioles present, fusoid, hyaline, thin-walled, basally swollen, with a sharp or often hyphoid neck, 15–22 × 4–4.5 μm; basidia clavate, hyaline, bearing four sterigmata and a basal clamp connection, 8.5–11 × 4.5–5 μm; basidioles similar in shape to basidia, but slightly shorter.

Basidiospores—Lunate, hyaline, thin-walled, smooth, occasionally with one to four guttules, IKI–, CB–, (3.5–)3.9–4.5(–4.8) × (0.7–) 0.8–1 μm, L = 4.22 μm, W = 0.93 μm, Q = 4.53 (*n* = 60/1).

Notes—*Sidera roseo-bubalina* was discovered in China, and the species is characterized by annual, resupinate basidioma with a pinkish buff to yellowish brown dry pore surface, round pores (6–7 per mm), a monomitic hyphal system and lunate basidiospores measuring 3.9–4.5 × 0.8–1 μm. Morphologically, *S. roseo-bubalina* and *S. lowei* share annual, resupinate basidioma and similar pores (6–7 per mm in *S. roseo-bubalina* vs. 6–8 per mm in *S. lowei* [1]), but *S. roseo-bubalina* has lunate basidiospores, which are usually <1 μm wide, while *S. lowei* has allantoid basidiospores, which are usually >1 μm wide. Phylogenetically, *S. roseo-bubalina* is close to *S. punctata* and *S. vesiculosa* (100% BS, 1.00 BPP; Figure 2), and these three species share annual and resupinate basidioma, and some generative hyphae with swollen tips, but *S. roseo-bubalina* has a pinkish buff to yellowish brown dry pore surface, while the pore surface is cream upon dryied in *S. vesiculosa* and cinnamon-buff to white when dried in *S. punctata*. In addition, *S. roseo-bubalina* differs from the other two species due to its larger pores (6–7 per mm in *S. roseo-bubalina*, 8–9 per mm in *S. punctata* and 7–9 per mm in *S. vesiculosa*, [7]).

5. ***Sidera vulgaris* sensu lato**

Specimens examined—Belarus, Brestskaya Voblasts, Belavezhskaya Pushcha National Park, on rotten wood of *Picea*, 19 October 2019, Y.C. Dai 21057 (BJFC 032716 and MSK). China, Guangxi Province, Guiping County, Xishan Scenic Spot, on rotten wood of *Pinus*, 25 December 2020, Y.C. Dai 22151 (BJFC 036043); Shannxi Province, Zhashui County, Niubeiliang Forest Park, on fallen angiosperm trunk, 16 September 2013, B.K. Cui 11216 (BJFC 015331). USA, Connecticut, New Haven, West Rock Park, on rotten stump of *Pinus*, 15 July 2012, Y.C. Dai 12730 (BJFC 013037).

Previously, the ten species of Sidera, viz. *S. lenis*, *S. lowei*, *S. lunata*, *S. minutipora*, *S. minutissima*, *S. parallela*, *S. srilankensis*, *S. tenuis*, *S. vesiculosa* and *S. vulgaris* were described or transferred to the genus. In this paper, *S. inflata*, *S. malaysiana*, *S. punctata* and *S. roseo-bubalina* were described as new to science, and they have resupinate, white to cream or buff fresh basidiocarps; a monomitic or dimitic hyphal system with generative hyphae bearing clamp connections; the presence of rosette-like crystals; and allantoid to lunate basidiospores. These characteristics fit well with the generic concept of Sidera. Thus far, 14 species are accepted in Sidera, and a key of accepted species is provided below.


**A key to species of Sidera worldwide**
1. Hymenium grandinioid to odontioid
*S. lunata*
1. Hymenium poroid22. Hyphal system monomitic32. Hyphal system dimitic63. Basidiospores mostly <1 μm in width43. Basidiospores mostly >1 μm in width54. Pores 7–9 per mm; basidiospores 2.9–3.7 μm long
*S. vesiculosa*
4. Pores 6–7 per mm; basidiospores 3.9–4.5 μm long
*S. roseo-bubalina*
5. Pores 6–8 per mm; cystidioles present, some branched 
*S. lowei*
5. Pores 8–9 per mm; cystidioles absent
*S. punctata*
6. Basidiospores >1.5 μm in width
*S. lenis*
6. Basidiospores <1.5 μm in width77. Skeletal hyphae becoming swollen in KOH87. Skeletal hyphae almost unchanged in KOH108. Pores 5–7 per mm; basidiospores 3.7–4.3 μm long
*S. minutipora*
8. Pores 9–11 per mm; basidiospores 2.9–3.3 μm long99. Basidiospores allantoid, skeletal hyphae distinctly swollen in KOH
*S. inflata*
9. Basidiospores lunate, skeletal hyphae slightly swollen in KOH
*S. malaysiana*
10. Tramal hyphae parallel along the tubes
*S. parallela*
10. Tramal hyphae interwoven1111. Generative hyphae at dissepiments even 
*S. srilankensis*
11. Generative hyphae at dissepiments with swollen tips1212. Basidiospores <3.6 μm long
*S. vulgaris*
12. Basidiospores >3.8 μm long1313. Sterile margin distinct, fimbriate; basidiospore Length/width <4
*S. minutissima*
13. Sterile margin indistinct to almost absent; basidiospore Length/width >4
*S. tenuis*


## 4. Discussion

As is well known, the genus *Sidera* is challenging for distinguishing species morphologically, and two species, *S. lenis* and *S. vulgaris*, were recognized before phylogenetical analyses.

*Poria krawtzewii* Pilát was treated as a synonym of *P. lenis* (P. Karst.) Sacc. [29], but it is different from the *S. lenis* complex because of ellipsoid spores [30]. We summarize another five synonyms of *Sidera lenis* (Index Fungorum and MycoBank): *Poria lunulispora* Pilát (type from Siberia), *P. chakasskensis* Pilát (type from Siberia), *P. earlei* Murrill (type from Jamaica), *P. tenuipora* Murrill (type from Jamaica) and *P. montana* Murrill (type from Jamaica).

*Poria earlei*, *P. montana* and *P. tenuipora* were described from the Caribbean (Jamaica) (Murrill [31,32]). The type of specimens of three species were studied by Niemelä and Dai [8]. They found that *P. earlei* and *P. montana* are conspecific, and the species is perennial, resupinate and has small pores (7–9 per mm). *Sidera punctata* and *S. vesiculosa* have similar pores (8–9 per mm in *S. punctata* vs. 7–9 per mm in *S. vesiculosa* [7]) to *P. tenuipora* as well, but the former two species have a monomitic hyphal system, while *P. tenuipora* has a dimitic hyphal system. *S. vesiculosa* differs from *S. punctata* due to the presence of vesicular cells of swollen hyphae in the subiculum.

*Poria chakasskensis* and *P. lunulispora* were described from Siberia [33,34]. Kotlaba and Pouzar [30,35] found that *P. chakasskensis* has basidiospores measuring 5.5–8.5 × 2–2.4 μm and represents *Ceriporia purpurea* (Fr.) Donk; *P. lunulispora* was collected on the wood of *Pinus*, and is true *Diplomitoporus lenis* (= *Sidera lenis*). Hence, *P. chakasskensis* and *P. lunulispora* are different from our newly described species.

Du et al. [2] summarized three synonyms of *Sidera vulgaris* (Index Fungorum and MycoBank): *Boletus papyraceus* Schrank, *B. proteus* Bolton and *B. cellulosus* O.F. Müll, and all of them were originally described from Europe. In addition, the specimen Ryvarden 37198 from New Zealand was named *S. vulgaris* by Miettinen and Larsson too [1]. In the present paper, we found three taxa with similar morphologies to *S. vulgaris*, but we did not study the types of the above-mentioned taxa, and no sequence data are available for them. Although our three taxa formed three distinct lineages in our phylogenies (Figure 3), we refrained from describing these as new, and treated them as *S. vulgaris* sensu lato in this paper (Table 2). The description of these species is the subject of a forthcoming paper.

*Ceriporiopsis lowei* (= *Sidera lowei*) was described from Northern Brazil [4]. The specimen Miettinen X426 (Ryvarden 38817) from New Zealand clustered together with a Venezuelan specimen Miettinen X419, and both specimens were considered as *Sidera lowei* by Miettinen and Larsson [1]. We did not examine the type of *Sidera lowei*; thus, we regard Miettinen X419 and Miettinen X426 as *“S. lowei”*.

The sequence of OTU1581 is from GenBank. We failed to obtain specimens of the taxa, but it formed a distinct lineage within the *Sidera* clade, so we treated OTU1581 as *Sidera* sp. temporarily here.

Polypores are an extensively studied group of Basidiomycota, and more than 1500 species have been recorded in the world [36,37,38,39,40,41,42]. Molecular phylogenies have demonstrated that more new taxa exist in the world [43,44,45,46,47], and more crypto species will be confirmed after molecular analyses of some traditional species in sensu lato. Thus, in order to understand the diversity, phylogeny and evolution of the fungi, future taxonomic and phylogenetic work should be based on both molecular and morphological characteristics.

## Figures and Tables

**Table 1:** The main characteristics of *Sidera* species. Pore and basidiospore sizes partly from Du et al. (2020) [2], Rajchenberg (1987) [4], Du et al. (2019) [7], Niemelä and Dai (1997) [8] and Niemelä (2005) [9].

Species	Growing Habit	Hymenophore	Hyphal System	Cystidioles	Skeletal Hyphae in KOH	Spores Shape	Spore Size (µm)
***S. inflata***	**Annual**	**Poroid, 9–10/mm**	**Dimitic**	**Present**	**Swollen**	**Allantoid**	**3–3.3 × 0.9–1.1**
*S. lenis*	Perennial	Poroid, 4–6/mm	Dimitic	Present	Swollen	Allantoid to lunate	3.9–4.9 × 1.5–2
*S. lowei*	Annual	Poroid, 6–8/mm	Monomitic	Present, some branched	-	Allantoid	3.5–5 × 1–1.2
*S. lunata*	Annual	Hydnoid, 8–9/mm	Monomitic	Present	-	Allantoid	2.5–3.8 × 1.6–1.9
***S. malaysiana***	**Annual**	**Poroid, 9–11/mm**	**Dimitic**	**Present**	**Swollen**	**Lunate**	**2.9–3.2 × 1–1.2**
*S. minutipora*	Annual	Poroid, 5–7/mm	Dimitic	Present	Swollen	Allantoid	3.7–4.3 × 1–1.3
*S. minutissima*	Annual	Poroid, 7–9/mm	Dimitic	Present	Almost unchanged	Allantoid	3.8–4.4 × 0.9–1.3
*S. parallela*	Annual	Poroid, 6–8/mm	Dimitic	Present	Almost unchanged	Lunate	2.8–3.3 × 0.9–1.2
***S. punctata***	**Annual**	**Poroid, 8–9/mm**	**Monomitic**	**Absent**	**-**	**Allantoid to lunate**	**3.8–4.8 × 1–1.3**
***S. roseo-bubalina***	**Annual**	**Poroid, 6–7/mm**	**Monomitic**	**Present**	**-**	**Lunate**	**3.9–4.5 × 0.8–1**
*S. srilankensis*	Annual	Poroid, 6–8/mm	Dimitic	Present	Almost unchanged	Lunate	3.5–4 × 1–1.3
*S. tenuis*	Annual	Poroid, 8–10/mm	Dimitic	Present	Almost unchanged	Allantoid	4.2–5 × 0.8–1
*S. vesiculosa*	Annual	Poroid, 7–9/mm	Monomitic	Present	-	Allantoid to lunate	2.9–3.7 × 0.6–1
*S. vulgaris*	Perennial	Poroid, 6–8/mm	Dimitic	Present, some branched	Almost unchanged	Allantoid to lunate	2.9–3.6 × 0.9–1.4

New species are shown in bold.

**Table 2:** Information for the sequences used in this study.

Species	Specimen No.	Locality	GenBank Accession No.						
			ITS	nLSU	RPB1	RPB2	TEF1	mtSSU	nSSU
*Ceriporiopsis aneirina*	MUAF 888	Czech Republic	EU340895	EU368503				EU368504	
*Contumyces rosella*	Redhead 7501		U66452	U66452					
*Exidia candida*	VS 8588	Russia	KY801871	KY801896			KY801920		
*Exidiopsis calcea*	MW 331	Canada	AF291280	AF291326					
*Gloeoporus dichrous*	KHL 11173	Norway	EU118627	EU118627					
*G. hainanensis*	Dai 15253	China	KU360402	KU360408					
*Globulicium hiemale*	Hjm 19007	Sweden	DQ873595	DQ873595					DQ873594
*Hyphodermella poroides*	Dai 12045	China	KX008367	KX011852					
*Odonticium romellii*	Murdoch 38	Finland	MF319073	MF318929					
*Oxyporus corticola*	KHL 13217	Estonia	DQ873641	DQ873641					DQ873640
*Repetobasidium conicum*	KHL 12338	USA	DQ873647	DQ873647					DQ873646
*Resinicium furfuraceum*	KHL 11738	Finland	DQ873648	DQ873648					
*Rickenella mellea*	Lamoure 74		U66438	U66438					
*Skvortzovia pinicola*	KHL 12224	USA	DQ873637	DQ873637					DQ873636
***Sidera inflata***	**Cui 13610**	**China**	MW198480 *						MW418088 *
*S. lenis*	Miettinen 11036	Finland	FN907914	FN907914					
“*S. lowei*”	Miettinen X419	Venezuela	FN907917	FN907917					
“*S. lowei*”	Miettinen X426	New Zealand	FN907919	FN907919					
*S. lunata*	JS 15063	Norway	DQ873593	DQ873593					
***S. malaysiana***	**Dai 18570**	**Malaysia**	MW198481 *	MW192007 *				MW424992 *	
*S. minutipora*	Gates FF257	Australia	FN907922	FN907922					
*S. minutipora*	Cui 16720	Australia	MN621349	MN621348	MW526261 *	MW505865 *	MW446248 *	MW424986 *	MW418078 *
*S. minutissima*	Dai 19529	Sri Lanka	MN621352	MN621350	MW526262 *			MW424994 *	MW418079 *
*S. minutissima*	Dai 18471A	China	MW198482 *	MW192008 *					
*S. parallela*	Cui 10346	China	MK346145					MW424991 *	MW418082 *
*S. parallela*	Cui 10361	China	MK346144						
***S. punctata***	**Dai 22119**	**China**	MW418438 *	MW418437 *					MW418085 *
***S. roseo-bubalina***	**Dai 11277**	**China**	MW198483 *						MW418081 *
*S.* sp.	OTU1581	Japan	MT594995						
*S. srilankensis*	Dai 19581	Sri Lanka	MN621345	MN621347	MW526265 *		MW427604 *	MW424993 *	MW418089 *
*S. srilankensis*	Dai 19654	Sri Lanka	MN621344	MN621346		MW505868 *	MW427602 *	MW424989 *	MW418087 *
*S. tenuis*	Dai 18697	Australia	MK331865	MK331867	MW526264 *	MW505866 *	MW427600 *	MW424988 *	MW418083 *
*S. tenuis*	Dai 18698	Australia	MK331866	MK331868		MW505867 *	MW427601 *	MW424985 *	MW418084 *
*S. vesiculosa*	BJFC025367	Singapore	MH636565	MH636567					
*S. vesiculosa*	BJFC025377	Singapore	MH636564	MH636566					
*S. vulgaris* sensu lato	Ryvarden 37198	New Zealand	FN907918	FN907918					
*S. vulgaris* sensu lato	Dai 12730	USA	MW198478 *						
*S. vulgaris* sensu lato	Dai 21057	Belarus	MW198484 *	MW192009 *		MW505869 *	MW427603 *	MW424987 *	MW418090 *
*S. vulgaris* sensu lato	Dai 22151	China	MW477794 *	MW474965 *				MW477795 *	
*S. vulgaris* sensu lato	Cui 11216	China	MW198485 *						
*Skeletocutis amorpha*	Miettinen 11038	Finland	FN907913	FN907913					
*S. chrysella*	Miettinen 9472	Finland	FN907916	FN907916					
*S. lilacina*	HHB 10522sp	USA	KY948834	KY948894					
*S. yuchengii*	FBCC 1132	China	KY953045	KY953045	KY953143		KY953109	KY953142	
*S. yunnanensis*	Dai 15709	China	KU950434	KU950436	MW526263 *		MW427605 *	MW424990 *	MW418080 *
*S. odora*	L 13763sp	Canada	KY948830	KY948893	KY949046				

* Newly generated sequences for this study. New species are shown in bold.

## Data Availability

Publicly available datasets were analyzed in this study. This data can be found here: https://www.ncbi.nlm.nih.gov/; https://www.mycobank.org/page/Simple%20names%20search; http://purl.org/phylo/treebase, submission ID 27909, 27910 and 27911.
